# Narrow-Gap Semiconductors and Low-Dimensional Structures for Optoelectronic Applications

**DOI:** 10.1155/2014/492521

**Published:** 2014-03-31

**Authors:** Fangyu Yue, Satyabrata Jit, Weida Hu

**Affiliations:** ^1^Key Laboratory of Polar Materials and Devices of MOE, East China Normal University, Shanghai 200241, China; ^2^Department of Electronics Engineering, Indian Institute of Technology (BHU), Varanasi 221005, India; ^3^National Laboratory for Infrared Physics, Shanghai Institute of Technical Physics, Chinese Academy of Sciences, Shanghai 200083, China

Advances in the growth, analysis, and characterization of various narrow-gap semiconductors and low-dimensional optoelectronic device structures have brought revolution into the modern day's information and communication technology including optical communication and computing systems. The main objective of this special issue is to include the recent advances in the experimental and theoretical research related to the growth, transport, and infrared properties of narrow-gap semiconductors and the fabrication, characterization, analysis, and simulation of various low-dimensional devices including quantum and nanostructures using narrow-gap semiconductors for optoelectronic applications. The basic aim of this special issue is to bring together in one publication the outstanding papers reporting new and original theoretical, experimental, and/or simulation works in the areas related to the narrow-gap semiconductors and low-dimensional structures.

Among a large number of submissions received from various researchers, we have selected only 7 papers for publication in this special issue. The work reported by H. Cui et al. is believed to be very useful in understanding the effects of metal-semiconductor contacts on the transient photovoltaic characteristics of HgCdTe-based photovoltaic arrays by using ultrafast lasers. In another paper, H. Cui et al. have analyzed the electrical modulation properties of the output intensity of two-photon absorption (TPA) pumping in HgCdTe photodiodes, which could be interesting for controlling a steady output intensity of TPA by adjusting electric field in HgCdTe-based photodetectors. Both papers of H. Cui et al. are highly important for the design, fabrication, and characterization of HgCdTe-based infrared photodetectors. The paper of D. P. Samajdar and S. Dhar reports a mathematical model for investigating the valence band structure of alloy systems InAs_1−*x*_Bi_*x*_ and InSb_1−*x*_Bi_*x*_ by using the concept of valence-band anticrossing technique. The theoretical results for band structure of InAs_1−*x*_Bi_*x*_ and InSb_1−*x*_Bi_*x*_ could be of immense importance for the designing of diluted Bi-doped narrow-gap InAs- and InSb-based mid-/long-wavelength optoelectronic devices. A two-dimensional simulation for the effects of donor-like surface traps on two-dimensional electron gas (2DEG) and drain current collapse of AlGaN/GaN high electron mobility transistors (HEMTs) has been investigated in detail by C. Yu et al. The paper of C. Zheng et al. reports the effects of same temperature GaN cap layer thickness on the quality of the InGaN quantum well material and the well/barrier interface of InGaN/GaN multiquantum-well based green light-emitting diodes grown on silicon substrates by MOCVD method. An interesting work on the phosphorescence of molecularly doped LEDs with blended polymer host and wide emission spectra reported by J. Wang et al. has also been considered for publication. The paper by R. Zhou et al. presents a detailed investigation on the second-harmonic generation (SHG), sum frequency generation (SFG), and difference frequency generation (DFG) from gold film with a periodic subwavelength air nanohole patterns by means of the three-dimension (3D) finite-difference time-domain (FDTD) algorithm, revealing the second-order nonlinearity in triangular lattice perforated gold film due to surface plasmas resonance.

The guest editors have tried their best to select only a few papers out of many covering both the theoretical and experimental state-of-the-art research in the area of this special issue. It is believed that this special issue will be of significant interests to the scientists and researchers working in the areas related to the narrow band gap semiconductors and low-dimensional optoelectronic structures.

